# Integrated in vivo and in vitro experiments with multi-omics analysis reveal SPP1 drives pancreatic cancer progression

**DOI:** 10.1186/s12885-026-15659-2

**Published:** 2026-02-24

**Authors:** Mujing Ke

**Affiliations:** 1 Department of Ultrasound, Xiangya Hospital, Central South University, Changsha, Hunan 410008 China; 2 National Clinical Research Center for Geriatric Diseases, Xiangya Hospital, Central South University, Changsha, Hunan 410008 China

**Keywords:** SPP1, Pancreatic cancer, Tumor immune microenvironment, Macrophage polarization, In vivo and in vitro experiments

## Abstract

**Objective:**

To investigate the expression pattern of SPP1 in pancreatic cancer and its role in the tumor immune microenvironment, with functional validation by in vivo and in vitro experiments.

**Methods:**

Bioinformatics analyses were performed using TCGA and CCLE databases to assess SPP1 expression, prognostic value, and immune correlations in pan-cancer and pancreatic cancer. Single-cell transcriptomic and CellChat analyses were used to explore cell communication and immune microenvironment characteristics. An orthotopic pancreatic cancer mouse model was established, and in vivo and in vitro experiments including flow cytometry, Western blot, co-immunoprecipitation (Co-IP), and in vivo ubiquitination assays were conducted to validate the immunoregulatory role of tumor-derived SPP1.

**Results:**

SPP1 was highly expressed in pancreatic cancer and associated with poor prognosis, elevated immunosuppressive microenvironment scores, and increased M2 macrophage infiltration. Single-cell and cell communication analyses indicated that SPP1 was mainly derived from macrophages and ductal epithelial cells, contributing to immune-regulatory signaling. Functional experiments confirmed that SPP1 promoted M2 macrophage polarization, enhanced immunosuppressive cytokine expression, and facilitated tumor progression through immune microenvironment remodeling.

**Conclusion:**

SPP1 plays a critical role in regulating macrophage polarization and shaping an immunosuppressive tumor microenvironment in pancreatic cancer, suggesting its potential as a therapeutic target.

**Supplementary Information:**

The online version contains supplementary material available at 10.1186/s12885-026-15659-2.

## Introduction

Pancreatic ductal adenocarcinoma (PDAC) is a highly malignant digestive system tumor with an extremely poor prognosis. In 2015, China reported 95,000 new cases of pancreatic cancer, ranking tenth among all malignancies, while the number of deaths reached 85,000, making it the fourth leading cause of cancer-related mortality [1]. With the rising global incidence and mortality of pancreatic cancer in recent years, it is projected to become the second leading cause of cancer-related deaths by 2030 [2]. PDAC exhibits occult onset and is difficult to diagnose early. Surgical resection remains the only potentially curative treatment, yet 80% of patients are already ineligible for surgery at diagnosis, and 80% of those who undergo resection still experience recurrence and distant metastasis [3–6].

The tumor microenvironment (TME) is composed of tumor cells, stroma, and infiltrating immune cells within tumor tissue [7]. Pancreatic cancer tissues are characterized by abundant stromal components that occupy a much larger volume than the tumor parenchyma, with extensive immune cell infiltration and tumor cells accounting for only approximately 10%−30%. The interaction between tumor cells and immune cells plays a crucial role in PDAC migration, invasion, metastasis, and chemotherapy resistance [8]. Macrophages coordinate immunosuppressive functions within the TME. Classified into M1 and M2 types, M1 macrophages promote inflammatory responses and exert anti-tumor effects, while M2 macrophages enhance immunosuppression and facilitate tumor progression [9–12]. Currently, macrophage-targeted therapy has emerged as a novel direction in anti-tumor immunotherapy. Therefore, investigating the pancreatic cancer microenvironment, particularly the mechanisms of macrophage function, may help improve patient prognosis.

Secreted phosphoprotein 1 (SPP1) is an important extracellular matrix component secreted by various cells, including tumor cells, fibroblasts, osteoclasts, and lymphocytes. As a key mediator of tumor-associated inflammation, SPP1 promotes cancer cell metastasis [13, 14]. SPP1 contains the classical arginine-glycine-aspartate (RGD) domain. The RGD site can bind to various integrins and CD44, regulating cell proliferation, adhesion, invasion, migration, and fibrosis [15–17]. SPP1 may serve as an emerging pan-cancer immunomodulatory factor and is closely associated with poor prognosis [18, 19]. Recent studies have shown that upregulated SPP1 expression in tumor tissues and plasma is associated with poor prognosis in various cancers, including breast, liver, and ovarian cancers [20–24]. Research indicates that SPP1 participates in TME formation, and colon cancer patients with SPP1-positive macrophages exhibit shorter progression-free survival (PFS) [25]. In lung adenocarcinoma, SPP1 can polarize macrophages toward the M2 phenotype [26]. An SPP1 + macrophage subpopulation is specifically enriched in the PDAC microenvironment, driving CD8 + T cell exhaustion through the SPP1-CD44 signaling axis to establish an immunosuppressive microenvironment [27]. However, how SPP1 regulates macrophage polarization and its downstream molecular mechanisms remain unclear.

This study combines bioinformatics analysis with experimental validation to demonstrate that SPP1 exhibits high expression characteristics in pancreatic cancer, with its expression levels significantly correlated with poor patient prognosis, elevated immunosuppressive microenvironment scores, and increased M2 macrophage infiltration. SPP1 is primarily derived from macrophages and ductal epithelial cells, functioning through participation in immune regulatory signaling networks. SPP1 promotes macrophage polarization toward the M2 phenotype, enhances the expression of immunosuppressive cytokines, and facilitates tumor progression by remodeling the immune microenvironment.

## Materials and methods

### Gene expression data download and preprocessing

Pan-cancer expression profile data were downloaded from the Xena database and transformed using log2(TPM + 0.01). The PAAD cohort with survival information was extracted for TCGA_PAAD-related analysis. Survival analysis was performed using corrected TCGA survival data [28]. Sample information for the TCGA-PAAD cohort is shown in Table 1. Gene expression RNA-seq data for cell lines were downloaded from the CCLE database (https://sites.broadinstitute.org/ccle). The pancreatic cancer single-cell dataset CRA001160, including transcriptomic data (H5 file) and metadata annotation files, was obtained from the TISCH database to construct a single-cell Seurat object.

**Table 1:** Dataset information from GEO database

TCGA PAAD cohort	Group information	Sample count
Status	Dead	93
	Alive	84
Age	Age > = 60	122
	Age < 60	55
Gender	Female	80
	Male	97
Stage	Stage I/II	167
	Stage III/IV	7
Grade	G1/2	125
	G3/4	50
Alcohol	No	64
	Yes	101

### Gene survival analysis

Kaplan–Meier analysis was used to generate survival curves, and the log-rank test was applied to determine statistical significance (p < 0.05 indicating significant survival differences between groups). Univariate Cox regression analysis was performed using the R packages survival or coxph function.

### Evaluation of immune cell infiltration proportions

Based on TCGA-PanCan expression profiles, immune cell infiltration proportions were calculated using CIBERSORT, ESTIMATE and ssGSEA (GSVA package) algorithms via the R package IOBR. The CIBERSORT algorithm [29] utilizes the LM22 signature matrix (547 leukocyte marker genes) to distinguish 22 immune cell types, including myeloid subsets, natural killer (NK) cells, plasma cells, naïve and memory B cells, and seven T-cell subsets. The sum of all immune cell proportions per sample equals 1. ssGSEA (GSVA package) evaluated the enrichment scores of 28 immune cell gene sets, representing the infiltration levels of these immune cell types.

### Single-cell transcriptome analysis

The R package Seurat was used to process single-cell expression matrices and metadata, including data normalization, dimensionality reduction, clustering, marker gene identification and visualization. Seurat object was constructed using CreateSeuratObject(), followed by cell annotation based on metadata. Marker genes were screened via FindAllMarkers() (threshold: p_val_adj < 0.05 & |avg_log2FC|> 0.5). For macrophage-specific analysis, a new Seurat object was generated after subsetting macrophage populations. Data normalization, scaling (top 2000 highly variable genes), PCA dimensionality reduction, and principal component selection via ElbowPlot() were performed. Cell subpopulations were identified using FindClusters(), followed by marker gene detection (FindAllMarkers()). Visualization was performed via VlnPlot(), DotPlot(), and functional enrichment analysis using compareCluster() (R package clusterProfiler).

### Cell–cell communication analysis

The CellChat package was employed to integrate ligand-receptor interaction databases (containing 2,021 experimentally validated pairs in humans and mice) and construct intercellular signaling networks from single-cell transcriptomic data.

### Tumor induction and sample preparation

Male C57BL/6 mice (Janvier Biolabs) were housed under SPF conditions. At 10–12 weeks of age, mice were anesthetized with ketamine/xylazine and injected with 5 × 10^5 Panc02 cells into the oral cavity floor. On day 21, mice were euthanized via cervical dislocation. Blood was collected via cardiac puncture, and spleens, tumor-draining lymph nodes (TDLNs), and tumors were harvested for histology and flow cytometry. Spleens and lymph nodes were mechanically dissociated through a 40-μm nylon mesh. Tumors were enzymatically digested using a Mouse Tumor Dissociation Kit (Miltenyi). Immune cells (CD45 +) were isolated using the EasySep™ Mouse Tumor-Infiltrating Lymphocyte Enrichment Kit (Stemcell Technologies). Flow cytometry was performed using 1 × 10^6 cells or 30 μL whole blood per sample.

### Flow cytometry

Cells were blocked with Fc receptor blocker (Miltenyi) and stained with FVS 700/780 viability dye (BD Biosciences). Surface antibody staining was performed on ice for 30 min. Blood samples were treated with lysis buffer (BD Biosciences) to remove RBCs. Samples were analyzed on a Cytek Aurora or Beckman Coulter Gallios flow cytometer, and data were processed using FlowJo v10.

### Co-immunoprecipitation (Co-IP)

Cell lysates were prepared using lysis buffer (Pierce, Rockford, USA). After centrifugation, lysates were pre-cleared with Protein A/G magnetic beads (Thermo Fisher Scientific) for 1 h at 4 °C. Pre-cleared lysates were incubated with primary antibodies overnight at 4 °C, followed by Protein A/G bead binding (1 h at 4 °C). Precipitated proteins were analyzed by Western blot.

### Intracellular ubiquitination assay

Cells were treated with 25 μM MG132 (proteasome inhibitor) for 8 h. After washing with PBS, cells were lysed in buffer containing 1% NP-40, 50 mM Tris–HCl (pH 7.4), 150 mM NaCl, 1 mM EDTA, 1 mM PMSF, protease inhibitors, and 20 mM N-ethylmaleimide (NEM) for 30 min on ice. Lysates were centrifuged (12,000 × g, 15 min, 4 °C), and supernatants were incubated with anti-Jun (or anti-Flag) antibody and Protein A/G beads (4 h at 4 °C). Immunoprecipitates were washed, boiled in SDS loading buffer, and analyzed by Western blot using an anti-ubiquitin antibody.

### Western blot

Proteins were extracted using RIPA buffer (Thermo Fisher Scientific, #89,901) with protease/phosphatase inhibitors (Roche, #4,693,159,001). Protein concentration was measured using a BCA assay kit (Thermo Fisher Scientific, #23,227). 20 μg protein/sample was separated on 10% SDS-PAGE and transferred to PVDF membranes (Millipore, #IPVH00010). Membranes were blocked in 5% non-fat milk/TBS-T (1 h, RT), then incubated with primary antibodies overnight at 4 °C: anti-Jun, anti-GAPDH (Abcam, #ab8245, 1:5000), and anti-Lamin (Cell Signaling Technology, #13,435, 1:1000). HRP-conjugated secondary antibodies (Cell Signaling Technology, #7074, 1:5000) were applied (1 h, RT). Protein signals were detected using ECL substrate (Thermo Fisher Scientific, #34,095) and imaged on a ChemiDoc system (Bio-Rad).

### Statistical analysis

All statistical analyses were performed using GraphPad Prism. Two-group comparisons used unpaired two-tailed Student's t-test. Multi-group comparisons used one-way ANOVA with Tukey's post hoc test. Kaplan–Meier survival curves were analyzed by log-rank test. Statistical significance was defined as* p* < 0.05.

## Results

### SPP1 High expression is associated with poor prognosis across multiple cancers

**Fig. 1:**
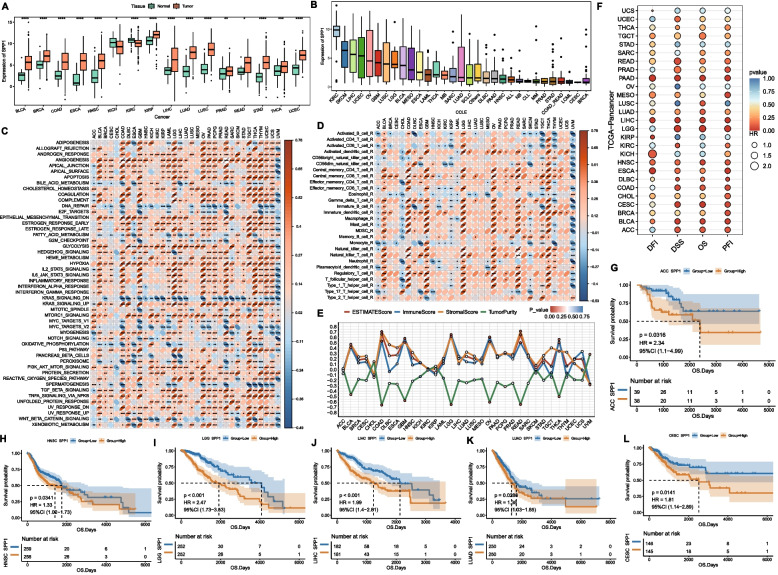
SPP1 expression landscape across pan-cancer types. **A** Boxplot of SPP1 expression differences between tumor (red) and normal tissues (green) in 16 cancer types (* indicates significant differences); **B** Boxplot of SPP1 expression across cancer cell lines from the CCLE database; **C** Heatmap of correlations between SPP1 expression and HALLMARK pathway enrichment scores; **D** Heatmap of correlations between SPP1 expression and infiltration levels of 28 immune cell types (color indicates correlation strength/direction; * indicates significance); **E** Line chart of correlations between SPP1 expression and four ESTIMATE scores; **F** Bubble plot of univariate Cox analysis for SPP1 based on OS, DSS, DFI, and PFI survival metrics; **G**-**L** Kaplan–Meier OS survival curves for high vs. low SPP1 expression groups

We first analyzed the differential expression of SPP1 between tumor and normal tissues in 16 solid tumors from the TCGA database. Results showed that SPP1 was significantly downregulated in KICH and KIRC but upregulated in most other tumors (Fig. 1A). Analysis of SPP1 expression in cancer cell lines from the CCLE database revealed high expression levels in KIRC, SKCM, LIHC, UCEC, and OV cell lines, while lower levels were observed in CESC, CLL, and STAD cell lines (Fig. 1B). Correlation analysis between SPP1 expression and HALLMARK gene set enrichment scores demonstrated negative associations with DNA repair, KRAS signaling downregulation, oxidative phosphorylation, and WNT/β-catenin signaling, but positive correlations with adipogenesis, angiogenesis, apoptosis, epithelial-mesenchymal transition (EMT), glycolysis, and hypoxia pathways (Fig. 1C).

A heatmap of immune cell infiltration proportions across cancers showed that SPP1 expression positively correlated with immune cell infiltration in most tumors, except for CHOL, UVM, and THYM, where negative correlations predominated (Fig. 1D). Notably, SPP1 expression exhibited a strong positive correlation with macrophage infiltration in multiple cancers. Additionally, SPP1 expression was positively associated with ESTIMATEScore, ImmuneScore, and StromalScore but negatively correlated with tumor purity, with the strongest correlations observed in COAD, LGG, GBM, READ, OV, and THCA (Fig. 1E).

Univariate Cox analysis based on OS, DSS, DFI, and PFI revealed that SPP1 expression significantly impacted survival in PAAD, LIHC, and LGG (Fig. 1F). Kaplan–Meier survival curves further confirmed that high SPP1 expression was associated with worse prognosis across multiple cancers (Fig. 1G–L), suggesting SPP1 as a pan-cancer prognostic biomarker.

### High SPP1 expression and its prognostic value in pancreatic cancer

**Fig. 2:**
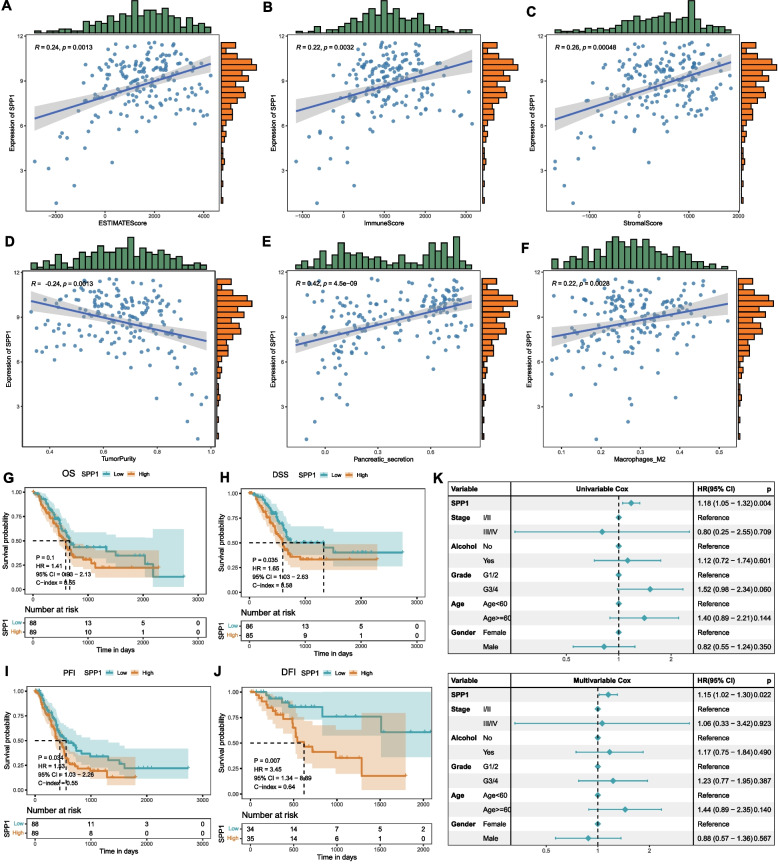
SPP1 expression and prognostic association in pancreatic cancer. **A**-**D** Scatter plots of SPP1 expression correlations with four ESTIMATE scores (R: correlation coefficient; p: significance); **E** Scatter plot of SPP1 expression correlation with pancreatic secretion pathway; **F** Scatter plot of SPP1 expression correlation with M2 macrophage infiltration (CIBERSORT); **G-J** Kaplan–Meier survival curves for OS, DSS, PFI, and DFI in the PAAD cohort; **K** Forest plot of univariate/multivariate Cox analysis for SPP1 combined with TCGA-PAAD clinical data

SPP1 expression showed significant positive correlations with ESTIMATE, immune, and stromal scores (p < 0.05) but a negative correlation with tumor purity (p < 0.05) (Fig. 2A–D). Pathway analysis indicated that SPP1 was positively associated with pancreatic secretion (Fig. 2E), suggesting its involvement in metabolic regulation. Immune infiltration analysis revealed a strong positive correlation between SPP1 expression and M2 macrophage infiltration (Fig. 2F), implicating its role in shaping an immunosuppressive TME.

Kaplan–Meier analysis demonstrated that high SPP1 expression predicted worse OS, DSS, DFI, and PFI (p < 0.05) (Fig. 2G–J). Univariate and multivariate Cox regression confirmed SPP1 as an independent prognostic factor (Fig. 2K).

### Single-cell expression landscape of SPP1 in pancreatic cancer

**Fig. 3:**
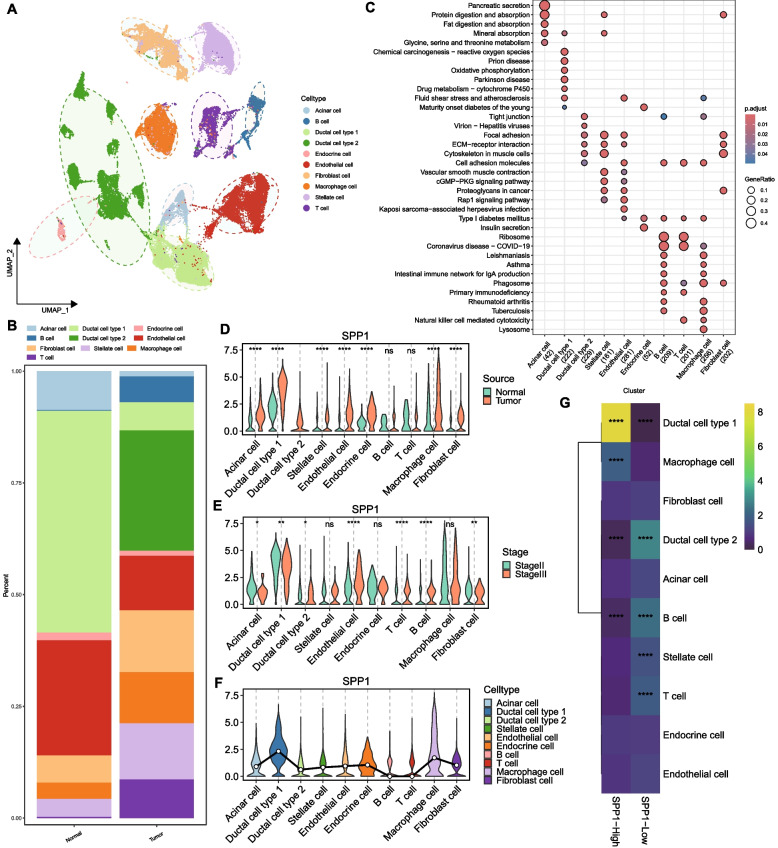
Single-cell profiling of SPP1 expression in the pancreatic tumor microenvironment. **A** UMAP annotation of pancreatic cancer single-cell data; **B** Stacked bar plot of cell type distribution in normal vs. tumor groups; **C** Bubble plot of KEGG enrichment for marker genes across cell types (dot size: proportion of marker genes in pathway; color: enrichment significance); **D** Violin plots of SPP1 expression differences across cell types in different tissues; **E** Violin plots of SPP1 expression across tumor stages; **F** Violin plots of SPP1 expression by cell type; **G** Heatmap of OR statistical analysis for SPP1 expression preference

Single-cell RNA-seq (scRNA-seq) analysis delineated the cellular heterogeneity of pancreatic cancer (Fig. 3A). Tumor samples exhibited increased proportions of B cells, T cells, and macrophages compared to normal tissues (Fig. 3B). KEGG enrichment analysis highlighted immune and metabolic pathways (Fig. 3C). SPP1 was upregulated in most tumor cell populations but relatively low in epithelial cells (Fig. 3D). Notably, SPP1 was highly expressed in type I ductal cells and macrophages (Fig. 3E–F). Odds ratio (OR) analysis confirmed type I ductal cells and macrophages as the primary sources of SPP1 (Fig. 3G).

### SPP1-mediated intercellular communication in pancreatic cancer

**Fig. 4:**
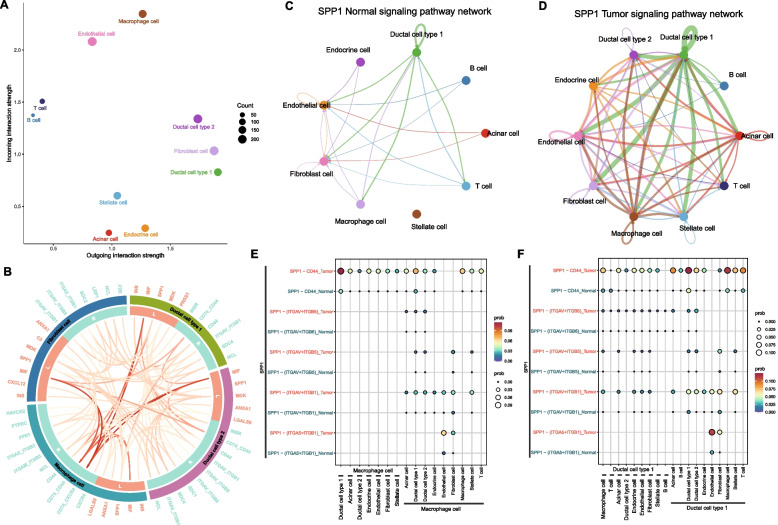
SPP1-mediated intercellular communication network in pancreatic cancer. **A** Scatter plot of cellular communication roles (x-axis: signal sending strength; y-axis: signal receiving strength); **B** Chord diagram of top active ligand-receptor pairs among ductal cells, fibroblasts, and macrophages; **C**-**D** Comparative communication networks of SPP1 signaling in normal vs. tumor groups; **E** Bubble plots of SPP1 signaling strength when macrophages act as receivers (left) or senders (right); **F** Bubble plots of SPP1 signaling strength when type I ductal cells act as receivers or senders

The use of CellChat for ligand-receptor communication analysis between macrophages, ductal cells, and fibroblasts illuminates the sophisticated intercellular crosstalk that orchestrates immunosuppressive networks in pancreatic tumors [30, 31]. CellChat analysis identified type I/II ductal cells and fibroblasts as major signal "senders," while macrophages acted as "receivers" (Fig. 4A). SPP1 signaling was markedly enhanced in tumors (Fig. 4C–D), particularly through SPP1-CD44 interactions between macrophages and ductal cells (Fig. 4E–F).

### Macrophage subpopulations expressing SPP1

**Fig. 5:**
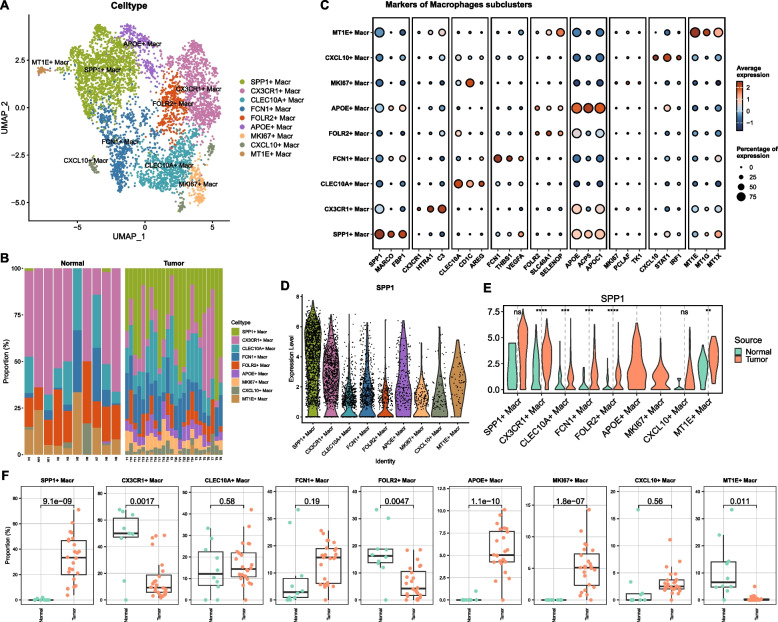
Characterization of macrophage subtypes and SPP1 expression patterns. **A** UMAP clustering of macrophage subtypes; **B** Stacked bar plot of macrophage subtype distribution in normal vs. tumor groups; **C** Bubble plot of marker gene expression for macrophage subtypes; **D** Violin plots of SPP1 expression across macrophage subtypes; **E** Violin plots comparing SPP1 expression in normal vs. tumor groups for each subtype; **F** Boxplot of proportional differences in macrophage subtypes between normal and tumor groups

Macrophages were clustered into nine subsets based on marker genes (SPP1, CX3CR1, CLEC10A, etc.) (Fig. 5A). Tumor tissues exhibited increased proportions of specific subsets (Fig. 5B–C). SPP1 + macrophages were significantly enriched in tumors (Fig. 5D–F).

### Functional characterization of SPP1 + macrophages

**Fig. 6:**
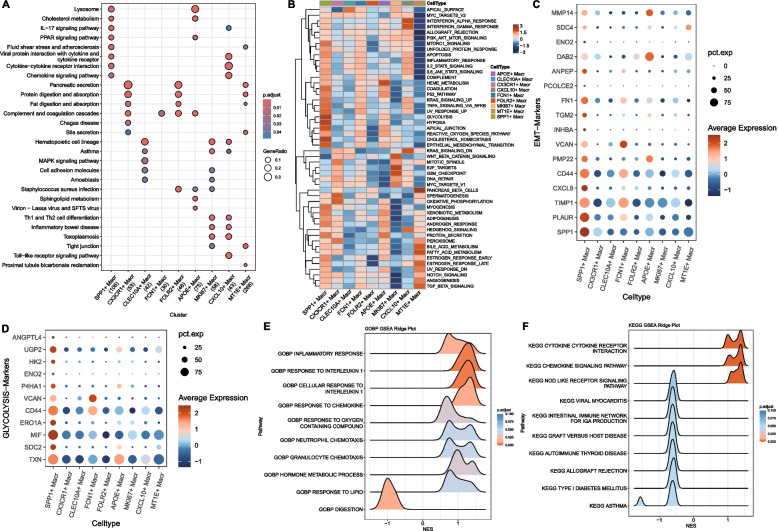
Functional features of SPP1 + macrophages in pancreatic cancer. **A** Bubble plot of KEGG enrichment for subtype marker genes (dot size: proportion of marker genes in pathway; color: enrichment significance); **B** Heatmap of HALLMARK ssGSEA scores based on subtype mean expression; **C**, **D** Bubble plots of EMT and glycolysis marker gene expression in SPP1 + macrophages (dot size: cell percentage; color: expression level); **E**, **F** GSEA mountain plots for differentially expressed genes in SPP1 + macrophages (NES > 0: tumor-enriched; NES < 0: normal-enriched)

SPP1 + macrophages were enriched in chemokine signaling, cytokine-receptor interaction, and PPAR pathways (Fig. 6A). Hallmark analysis revealed activation of EMT, glycolysis, hypoxia, and ROS pathways (Fig. 6B–D). GSEA showed upregulation of inflammatory responses and cytokine signaling in tumors (Fig. 6E–F).

### OPN promotes tumor growth by suppressing CD8 + T cells

**Fig. 7:**
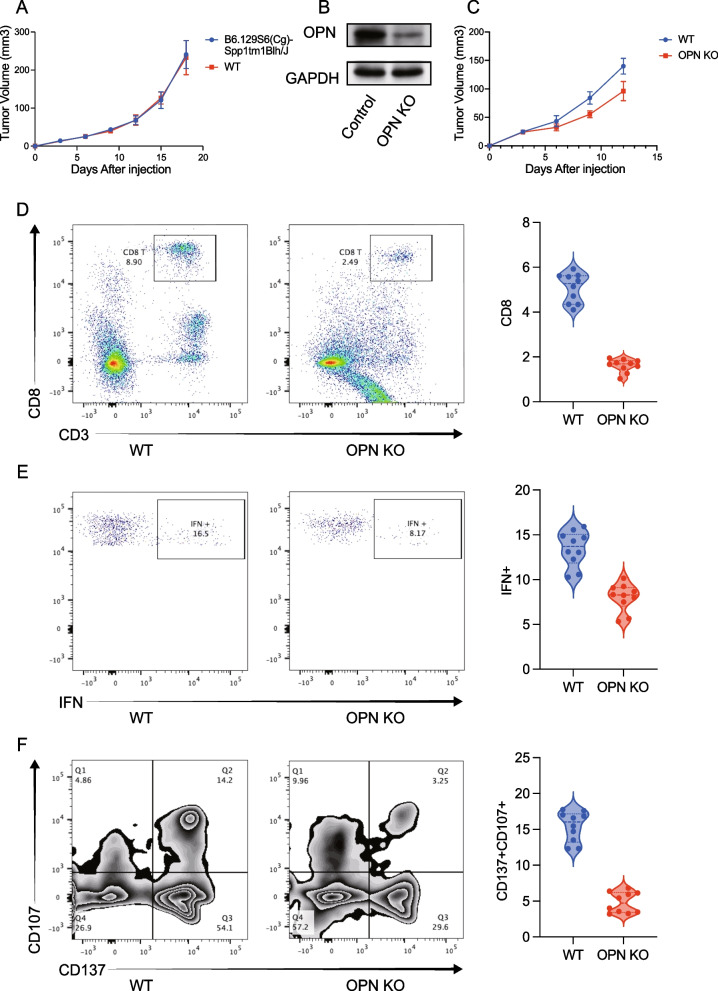
Tumor-derived OPN suppresses antitumor immunity and promotes tumor progression. **A** Tumor growth in B6.129S6(Cg)-Spp1tm1Blh/J (OPN knockout, KO) mice showed no significant difference compared to wild-type controls. **B** Establishment of OPN KO tumor cell line. **C** Tumor growth was significantly reduced when using OPN KO tumor cells, suggesting that tumor-derived OPN promotes tumor progression. **D** Infiltration of CD8 T cells in the tumor microenvironment. **E** Expression of IFN-γ by tumor-infiltrating CD8 T cells. **F** Activation of CD8 T cells assessed by expression of CD137 and CD107a

In vivo studies using OPN-knockout mice showed no significant change in tumor volume (Fig. 7A–B). However, OPN-overexpressing tumors exhibited accelerated growth (Fig. 7C). Flow cytometry revealed reduced CD8 + T cell infiltration and impaired IFN-γ production in OPN-high tumors (Fig. 7D–E).

### OPN drives tumor progression via macrophage polarization

**Fig. 8:**
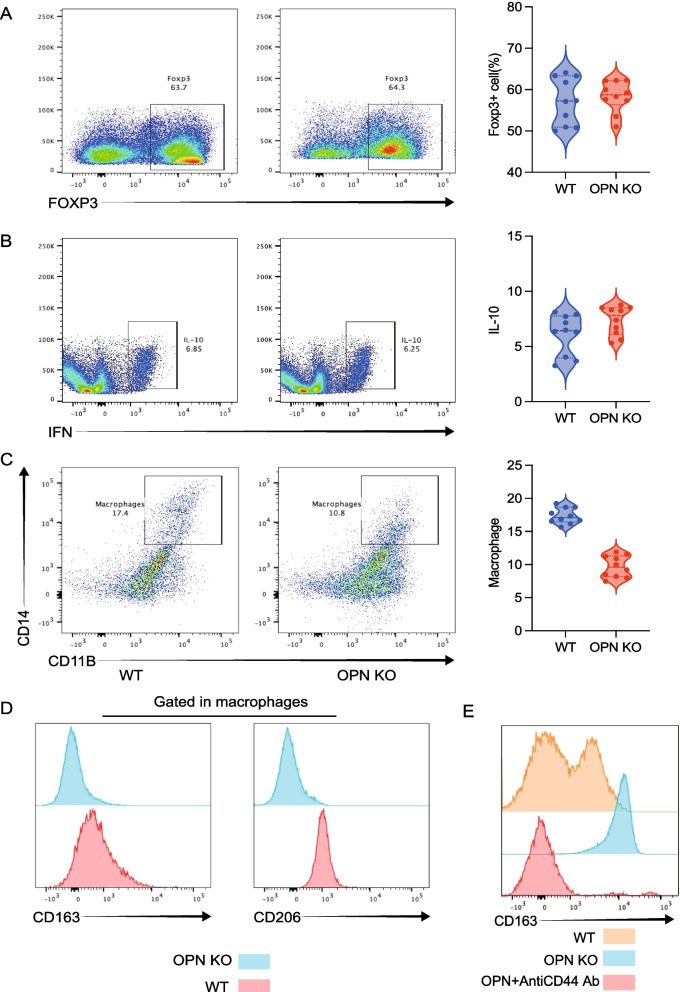
Tumor-derived OPN promotes macrophage polarization without affecting Treg cells. **A** Infiltration levels of FOXP3⁺ CD4 T regulatory cells. **B** IL-10 secretion by CD4⁺ T cells in PBMCs was not affected after OPN knockdown. **C** Tumor-infiltrating macrophages (CD14⁺CD11b⁺) were significantly reduced following OPN knockdown. **D** Expression of M2 macrophage markers (CD206, CD163) was significantly decreased upon OPN knockdown. **E** OPN was used to induce macrophages polarization, anti-CD44 Ab was used to block CD44 function. CD163 was evaluated by flowcytometry

**Fig. 9:**
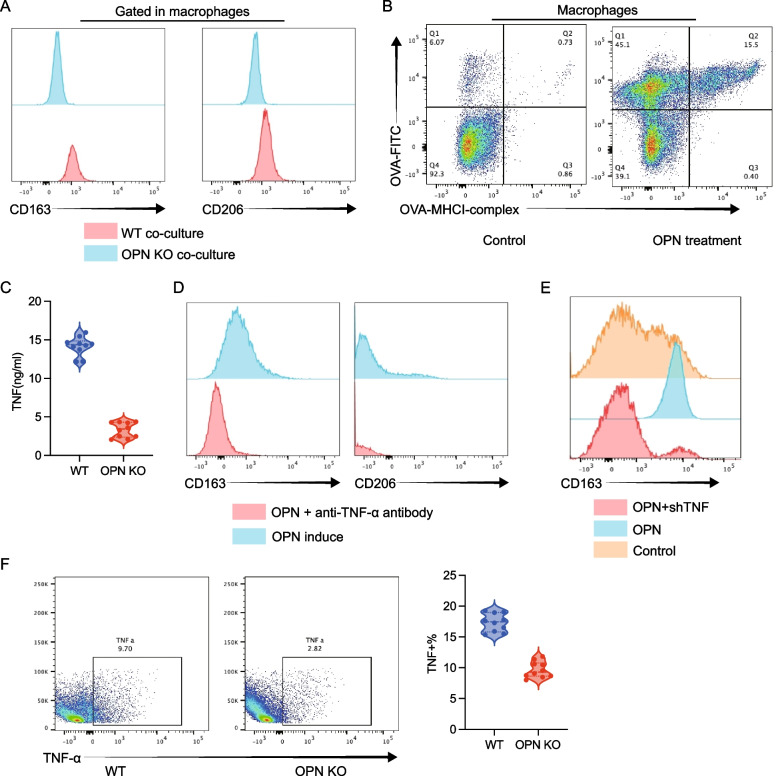
OPN promotes macrophage polarization via enhancing TNF-α autocrine signaling. **A** In vitro co-culture of macrophages with WT or OPN KO tumor cells showed reduced M2 marker expression (CD206, CD163) in the OPN KO group. **B** Macrophages with or without received OPN induction was co-cultured with OVA-FITC to evaluate engulfment function. Antibody against OVA-MHCI complex was used to evaluate antigen-presentation function. **C** ELISA demonstrated that TNF-α secretion was significantly reduced in macrophages co-cultured with OPN KO tumor cells. **D** OPN-stimulated macrophages showed increased M2 marker expression, which was markedly attenuated when co-treated with anti-TNF-α antibody. **E** OPN-stimulated macrophages showed increased M2 marker expression, which was markedly attenuated when TNF was knocked down. **F** Tumor-infiltrating macrophages in OPN KO tumors expressed lower levels of TNF-α

OPN knockdown did not affect Tregs (Fig. 8A–B) but significantly decreased tumor-associated macrophages (TAMs) and M2 markers (CD163/CD206) (Fig. 8C–D). Given single cell analysis result shown CD44-SPP1 axis is important during crosstalk, we investigated this axis in vitro. as shown in Fig. 8E, OPN treatment promotes CD163 expression in macrophages, but antibody against CD44 blocked this function. Indicated CD44 is required for OPN induced effect. OPN-deficient tumor cells failed to induce M2 polarization (Fig. 9A). We further confirmed influence of OPN treatment on macrophages. As shown in Fig. 9B, OPN treatment increased engulfment function and antigen presentation function of macrophages. Exogenous OPN promoted CD163/CD206 expression, which was attenuated by TNF neutralization (Fig. 9D).We further confirmed this result by knock down of TNF (Fig. 9E). TNF expression level was decreased when co-cultured with tumor cell with SPP1 KO(Fig. 9F).

### OPN regulates TNF autocrine signaling via Itch-Jun axis

**Fig. 10:**
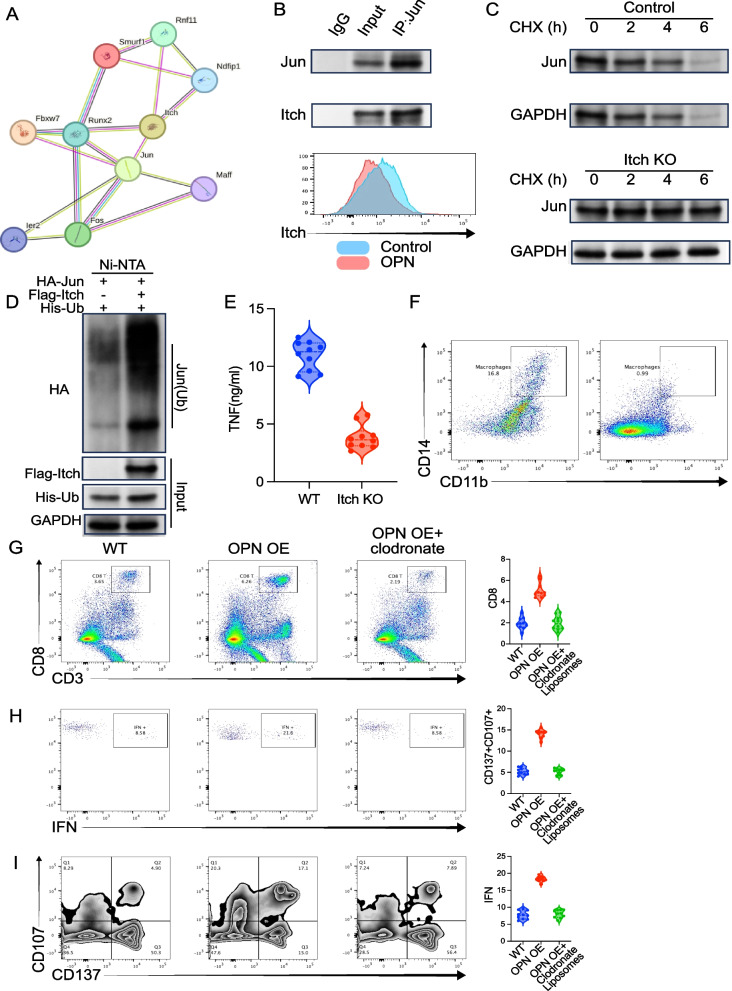
Itch regulates TNF-α autocrine signaling by promoting Jun ubiquitination. **A** Jun, a known upstream regulator of TNF-α, is predicted to interact with Itch. **B** Top: Immunoprecipitation assay shows Jun interacts with Itch; Bottom: OPN treatment increases Itch expression. **C** Jun degradation is delayed under Itch knockdown conditions following CHX treatment. **D** In vitro ubiquitination assay demonstrates that Itch promotes Jun ubiquitination. **E** TNF-α expression is reduced upon Itch knockdown under LPS stimulation. **F** Clodronate liposomes effectively deplete tumor-infiltrating macrophages (CD14⁺CD11b⁺). **G** Infiltration of CD8 T cells under different conditions: WT, OPN overexpression, and OPN overexpression plus clodronate liposomes. **H** IFN-γ expression by tumor-infiltrating CD8 T cells under the same conditions. **I** Expression of activation markers CD137 and CD107a on CD8 T cells under the same conditions

OPN upregulated Itch expression, which promoted Jun ubiquitination and degradation (Fig. 10A–D). Itch overexpression increased TNF secretion (Fig. 10E). Macrophage depletion abolished OPN-mediated CD8 + T cell suppression (Fig. 10F–I), confirming their pivotal role in OPN-driven immune evasion. Gating strategy of tumor-infiltrated CD8 + T cell or macrophages was shown in Supplement Fig. 1.

## Discussion

In recent years, an increasing number of studies have employed multi-omics approaches integrating TCGA, CCLE, and single-cell transcriptomics to combine bioinformatics analysis with refined biological validation, aiming to explore key genes and cellular expression patterns that drive malignant tumor progression [32–35]. This study systematically elucidates the key mechanistic roles and clinical significance of SPP1 in pancreatic cancer through multi-omics analyses. Pan-cancer analysis confirmed that high SPP1 expression is significantly associated with poor prognosis in 12 malignancies. In pancreatic cancer, while SPP1 expression strongly correlates with immune scores, it coincides with CD8 + T cell functional suppression, suggesting that SPP1 may exert its effects through unique immunomodulatory mechanisms. Existing studies indicate that SPP1 is closely linked to immune responses. In ovarian cancer, SPP1 expression levels are associated with immune infiltration, including macrophages, dendritic cells, mast cells, and CD4 + T cells [23].

With advancements in single-cell sequencing technology, growing evidence highlights the heterogeneity of macrophages in the tumor microenvironment (TME). SPP1 + macrophages have been identified in various cancers, including breast cancer, colorectal cancer, and PDAC [26, 36–38]. In gastric cancer, crosstalk between SPP1 + tumor-associated macrophages (TAMs) and exhausted CD8 + T cells fosters an immunosuppressive microenvironment, enhancing co-inhibitory receptor expression and further promoting CD8 + T cell apoptosis, thereby reducing the efficacy of immunotherapy [39]. In colorectal cancer, macrophages with high SPP1 expression exhibit poor responses to anti-PD-L1 therapy, likely due to their predominant localization in tumor tissues and their synergistic interaction with fibroblasts, which may promote desmoplasia and hinder immunotherapy [25]. Our single-cell analysis revealed that SPP1 + macrophage subsets are specifically enriched in the pancreatic cancer TME and are significantly associated with pro-tumorigenic pathways. This finding is consistent with previously published literature. Song et al. demonstrated that TAMs can suppress antitumor immunity by modulating their own metabolism. Integrating our results, we hypothesize that SPP1 + TAMs constitute a central hub linking metabolic dysregulation, tumor progression, and immune escape [40]. In vivo and in vitro experiments further demonstrated that OPN knockdown selectively reduces M2 macrophage infiltration, an effect that disappears upon macrophage depletion, confirming macrophages as the key mediators of SPP1-driven immune regulation. Recent studies further support this view [41]. SPP1 not only acts as a driver of macrophage polarization but also serves as a central signaling hub connecting cancer cells, fibroblasts, and immune cells. Its overexpression can lead to a comprehensive immunosuppressive state and therapy resistance. This discovery provides new insights into the immunosuppressive TME of pancreatic cancer, suggesting that SPP1 may indirectly induce CD8 + T cell exhaustion by shaping an M2 macrophage-dominated immune landscape.

Previous studies have shown that TAM-derived SPP1 modulates the immune microenvironment in hepatocellular carcinoma (HCC). SPP1 inhibitors or anti-CD44 antibodies can reduce tumor burden and restore T cell function [42]. SPP1 promotes malignant progression by interacting with various receptors on tumor cell surfaces [43]. In intrahepatic cholangiocarcinoma, the SPP1-CD44 axis drives immunosuppression, impairing T cell proliferation and correlating with poor prognosis [44]. Our findings further validate the critical role of the SPP1-CD44 signaling axis in intercellular communication within pancreatic cancer, suggesting that targeting this axis may represent a potential immunotherapeutic strategy. Notably, our study revealed enhanced SPP1 signaling activity in tumor tissues, particularly in the crosstalk between type I ductal cells and macrophages, where SPP1-CD44 interactions play a prominent role.

To further explore the mechanisms by which SPP1 regulates macrophages to promote pancreatic cancer progression, prior studies have shown that SPP1-expressing TAMs exhibit pro-tumorigenic properties by reducing phagocytic activity and enhancing angiogenesis, thereby facilitating tumor growth [45, 46]. Delving deeper into SPP1's oncogenic mechanisms, we discovered that Itch not only promotes Jun protein ubiquitination and degradation but also increases TNF secretion upon overexpression, forming an "SPP1-CD44-Itch-Jun-TNF" feedback loop. This study is the first to identify Itch as a downstream effector mediating Jun ubiquitination in this pathway. Intriguingly, the observed reduction in TNF autocrine signaling contrasts with the classical pro-inflammatory role of TNF, suggesting that SPP1 may fine-tune TNF secretion thresholds to achieve immunosuppression. This finding provides novel theoretical insights into the complex regulation of cytokine networks in the TME.

From a clinical translation perspective, this study holds significant therapeutic implications. The proportion of SPP1 + macrophages combined with ESTIMATE scores may serve as a novel diagnostic biomarker for immune subtyping in pancreatic cancer, while small-molecule inhibitors targeting Itch could represent a potential strategy to reverse SPP1-mediated immunosuppression. The development of SPP1-targeted drugs in combination with existing PD-1 antibodies offers broad clinical prospects. This study not only systematically elucidates the multi-faceted mechanisms of SPP1 in pancreatic cancer but also provides new intervention strategies and therapeutic targets to overcome current challenges in immunotherapy resistance.

However, this study has certain limitations that should be noted. Further validation, including mapping specific ubiquitination sites on Jun protein, conducting CRISPR-mediated genetic rescue experiments, and employing E3 ligase-dead Itch mutants, remains technically challenging in primary macrophage systems. While this study incorporated animal experiments, it remains challenging to fully replicate the complex immune microenvironment of human PDAC. Furthermore, the cellular sources of SPP1 identified primarily through transcriptomics require spatial protein-level validation in human tissues. The global transcriptional reprogramming of macrophages induced by SPP1, the quantitative dynamics of the proposed TNF-Jun feedback loop, and the direct role of SPP1 in immune cell chemotaxis need further elucidation through omics profiling, mathematical modeling, and directed migration assays, respectively. Finally, the therapeutic strategies targeting SPP1 or Itch suggested by our findings remain hypothetical; their translational potential must be rigorously evaluated through independent pharmacological development and combination therapy testing in subsequent studies. These limitations clarify critical gaps between mechanistic discovery and clinical translation, providing a clear roadmap for future research.

## Supplementary Information

Supplementary Material 1.

Supplementary Material 2.

## Data Availability

The datasets used and/or analysed during the current study are available from the corresponding author on reasonable request.

## Abbreviations

Co-IP: Co-immunoprecipitation
PDAC: Pancreatic ductal adenocarcinoma
TME: The tumor microenvironment
SPP1: Secreted phosphoprotein 1
RGD: Arginine-glycine-aspartate
PFS: Progression-free survival
NK: Natural killer
HCC: Hepatocellular carcinoma
