# Supplementary figures and images for: Integrated in vivo and in vitro experiments with multi-omics analysis reveal SPP1 drives pancreatic cancer progression
Source: BMC Cancer. 2026 Feb 24;26:419. doi: 10.1186/s12885-026-15659-2 (PMC13036904; doi:10.1186/s12885-026-15659-2)

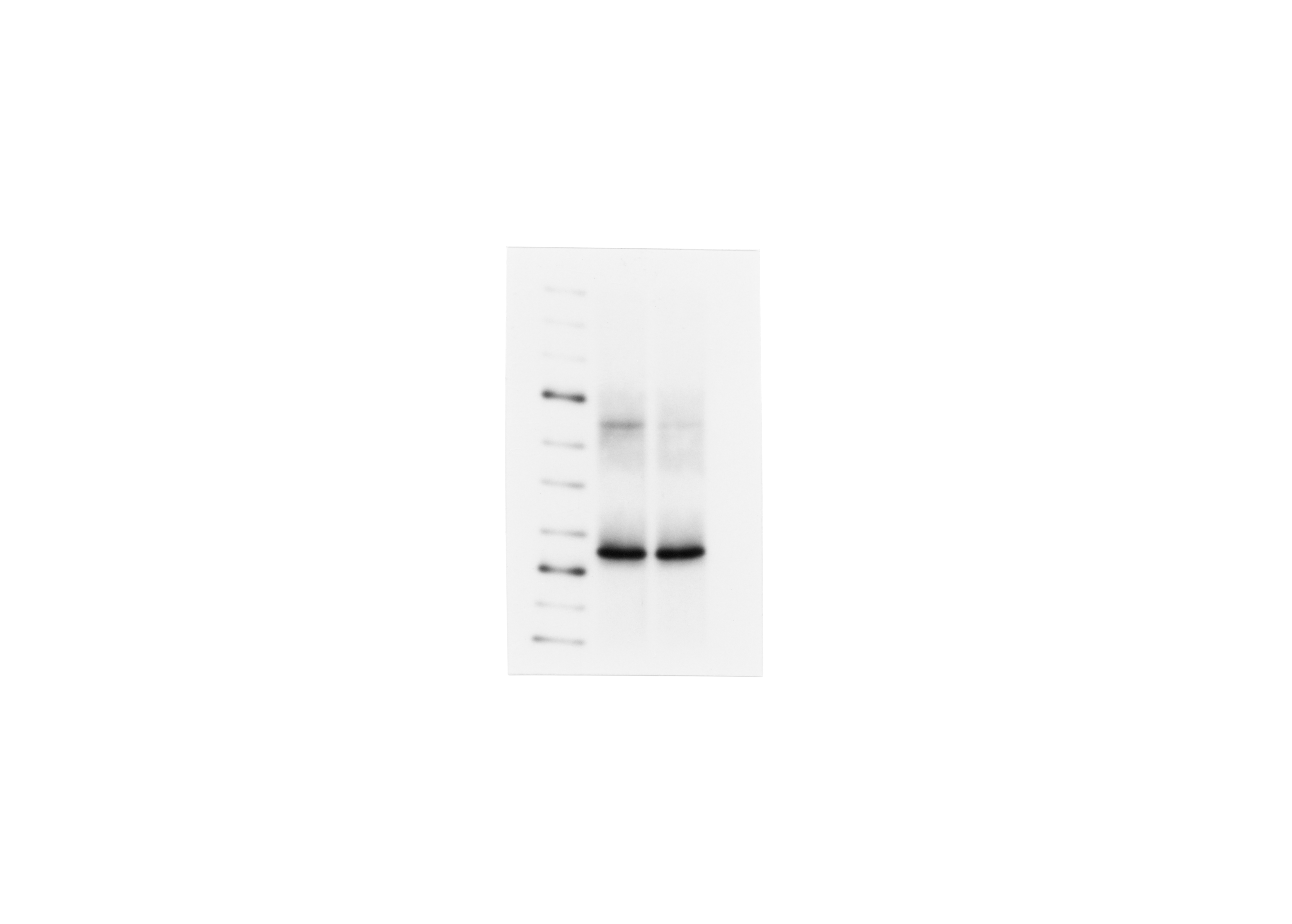

Supplement: Supplementary file 1 — Supplementary Material 1. [file 12885_2026_15659_MOESM1_ESM.zip › original westernblot data/1/Fig7-GAPDH.tif]

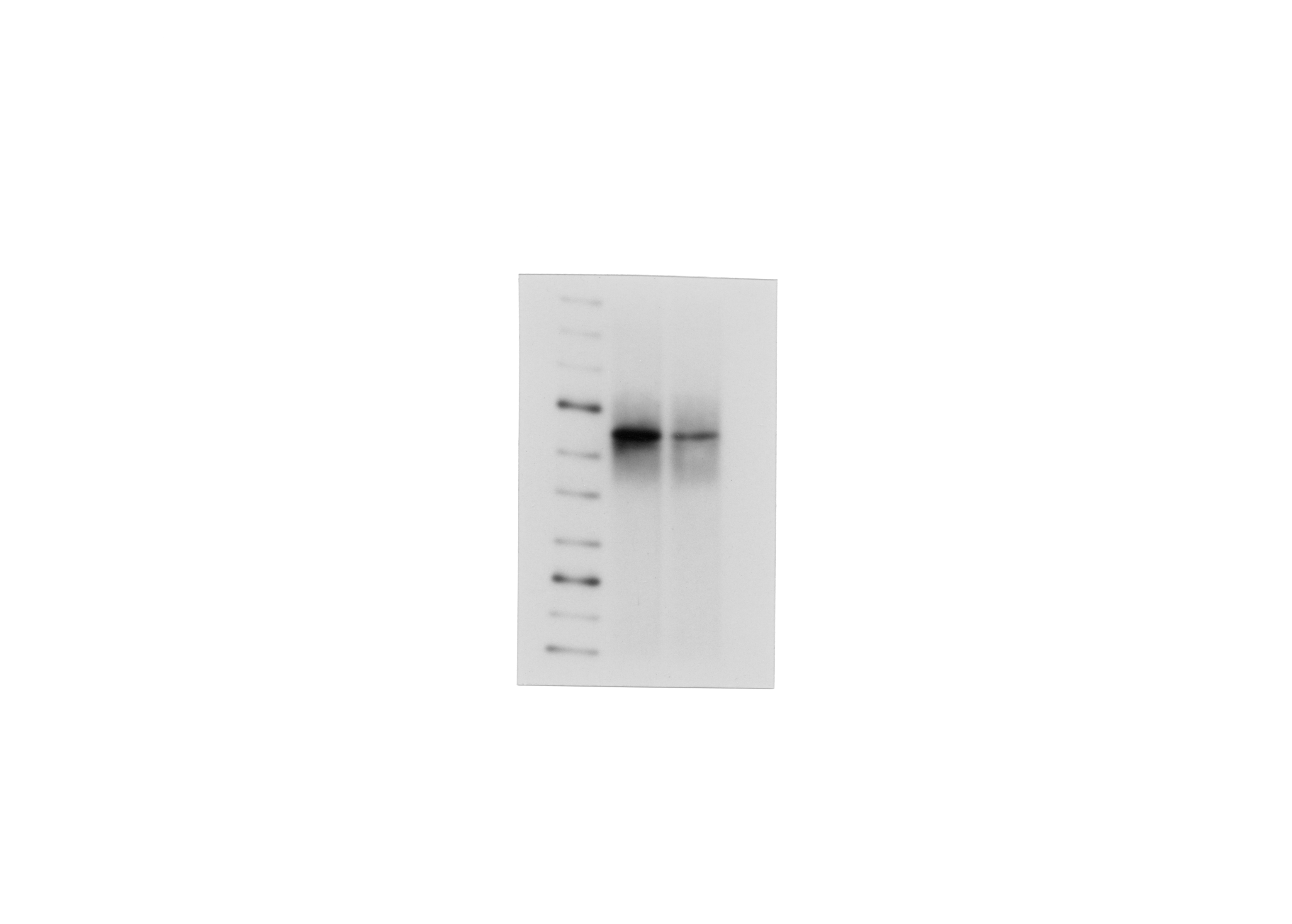

Supplement: Supplementary file 1 — Supplementary Material 1. [file 12885_2026_15659_MOESM1_ESM.zip › original westernblot data/1/Fig7-OPN.tif]

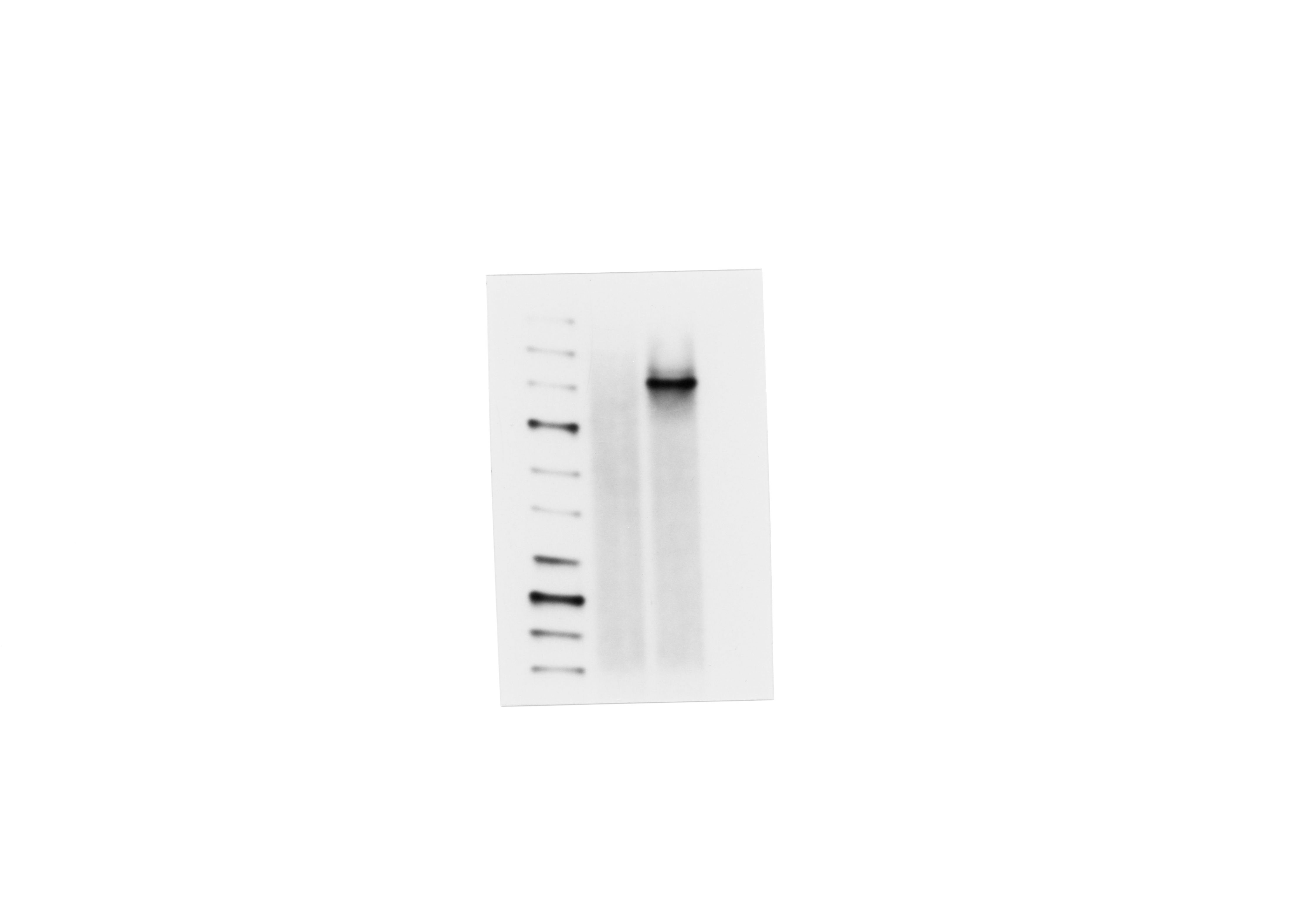

Supplement: Supplementary file 1 — Supplementary Material 1. [file 12885_2026_15659_MOESM1_ESM.zip › original westernblot data/2/Fig10D-Flag-ltch.tif]

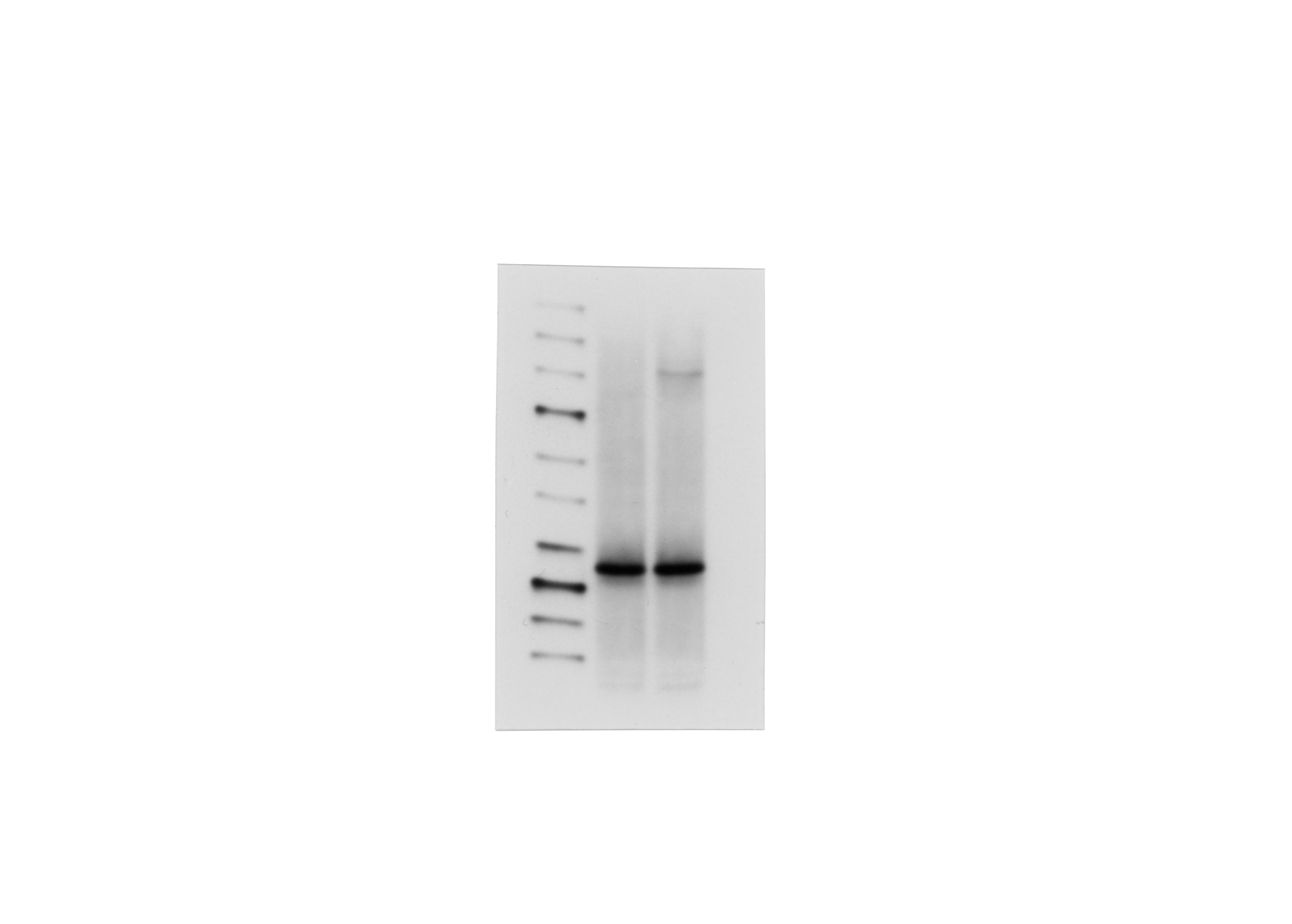

Supplement: Supplementary file 1 — Supplementary Material 1. [file 12885_2026_15659_MOESM1_ESM.zip › original westernblot data/2/Fig10D-GAPDH.tif]

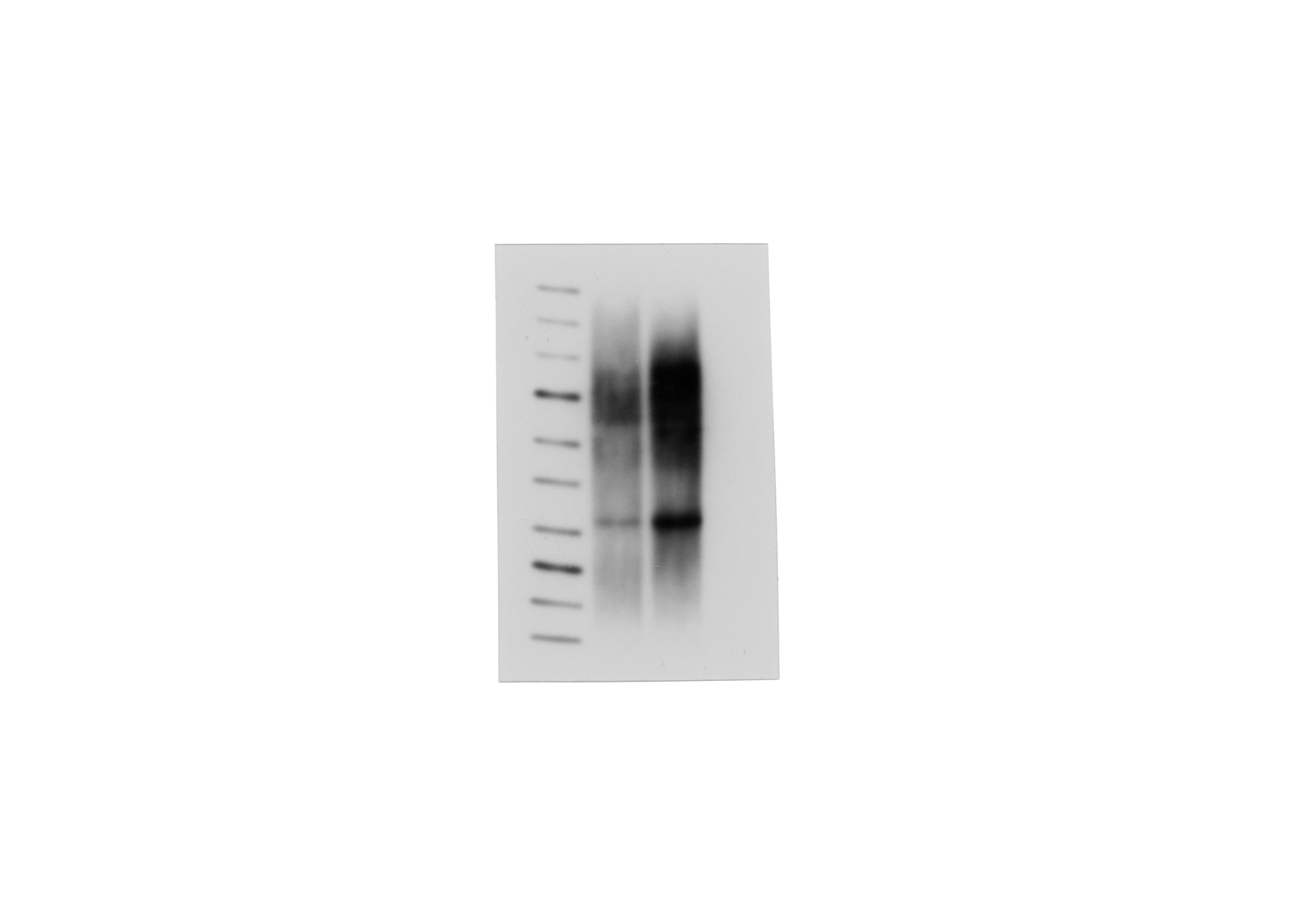

Supplement: Supplementary file 1 — Supplementary Material 1. [file 12885_2026_15659_MOESM1_ESM.zip › original westernblot data/2/Fig10D-HA.tif]

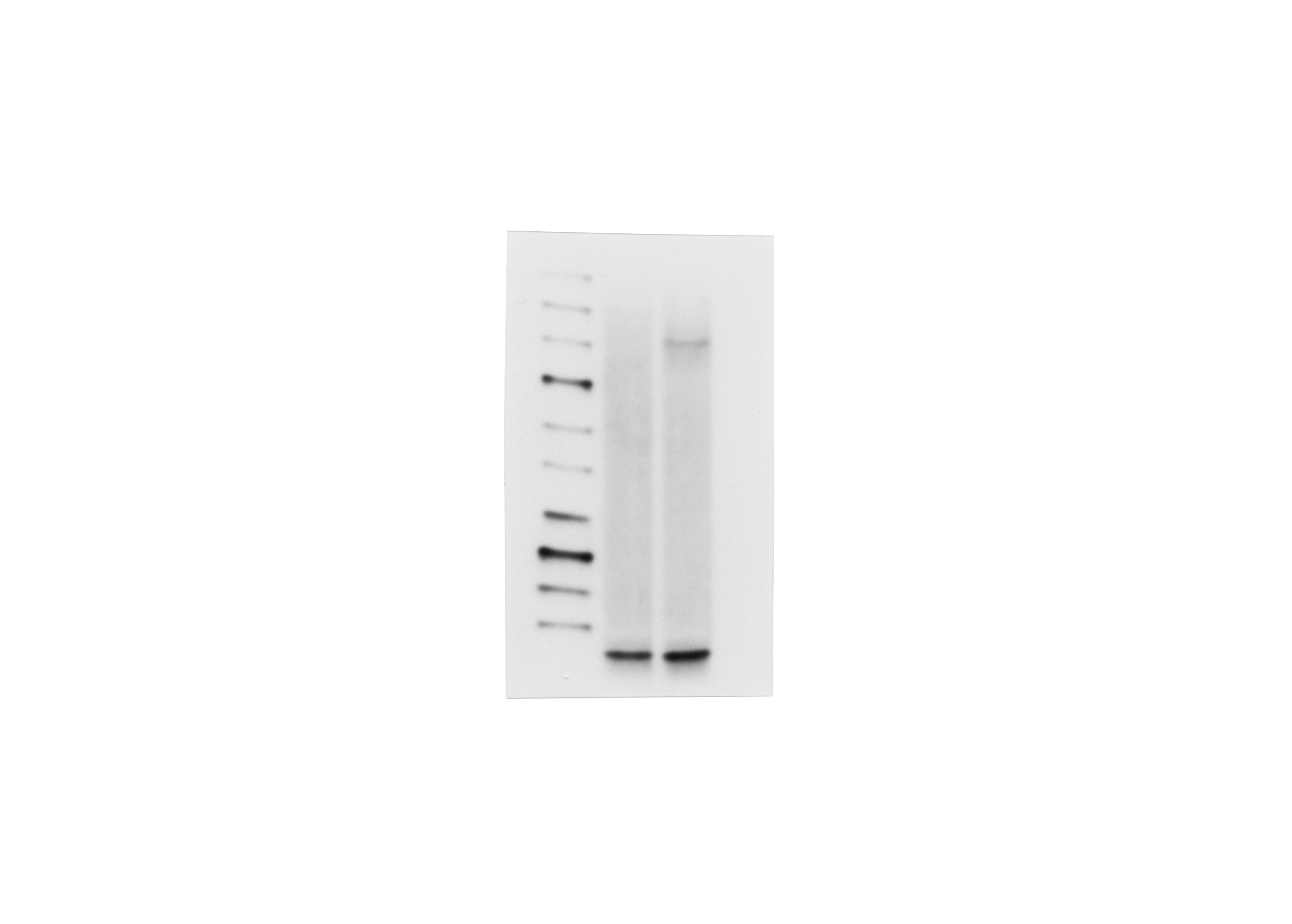

Supplement: Supplementary file 1 — Supplementary Material 1. [file 12885_2026_15659_MOESM1_ESM.zip › original westernblot data/2/Fig10D-His-Ub.tif]

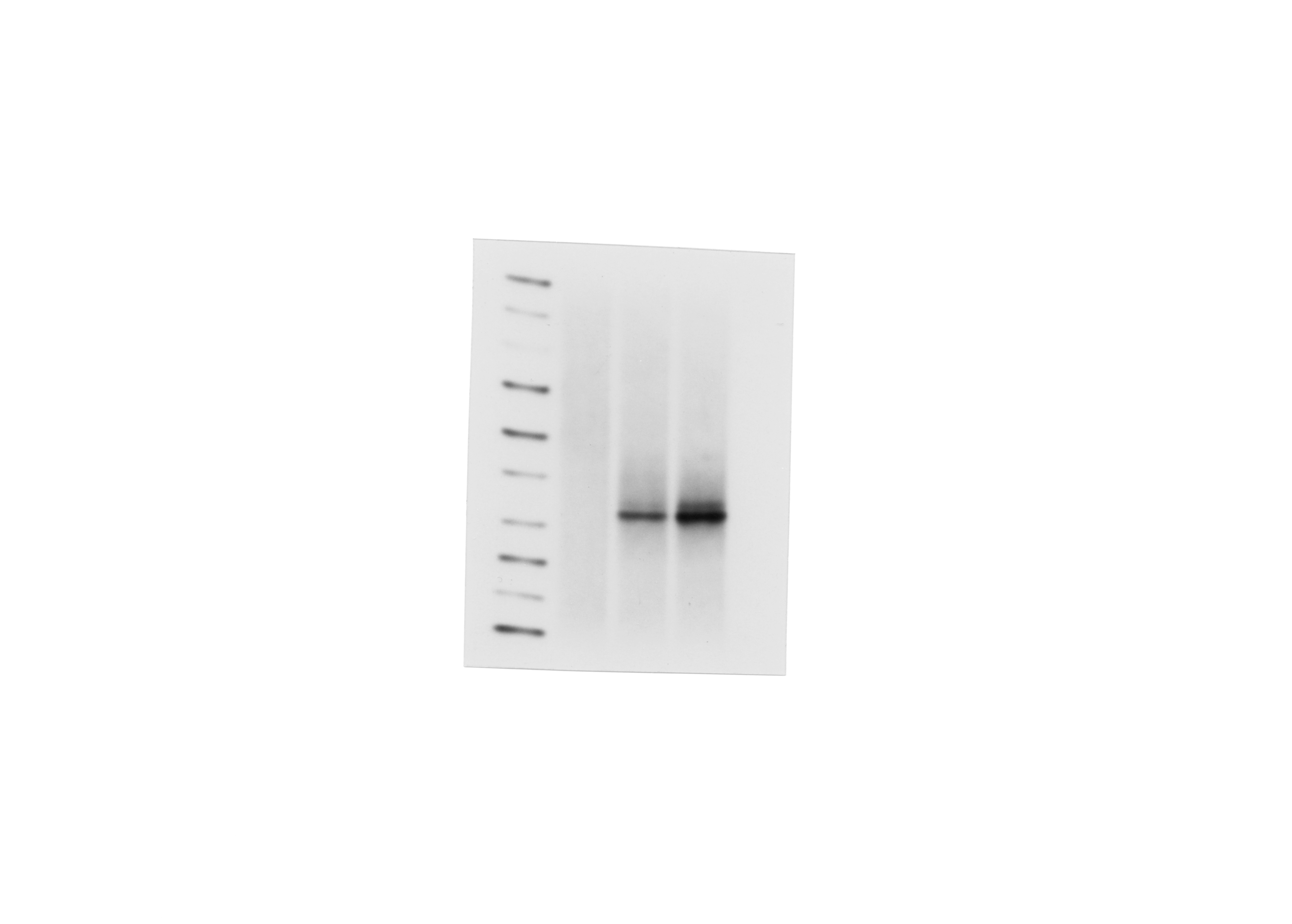

Supplement: Supplementary file 1 — Supplementary Material 1. [file 12885_2026_15659_MOESM1_ESM.zip › original westernblot data/3/Fig10B-Jun.tif]

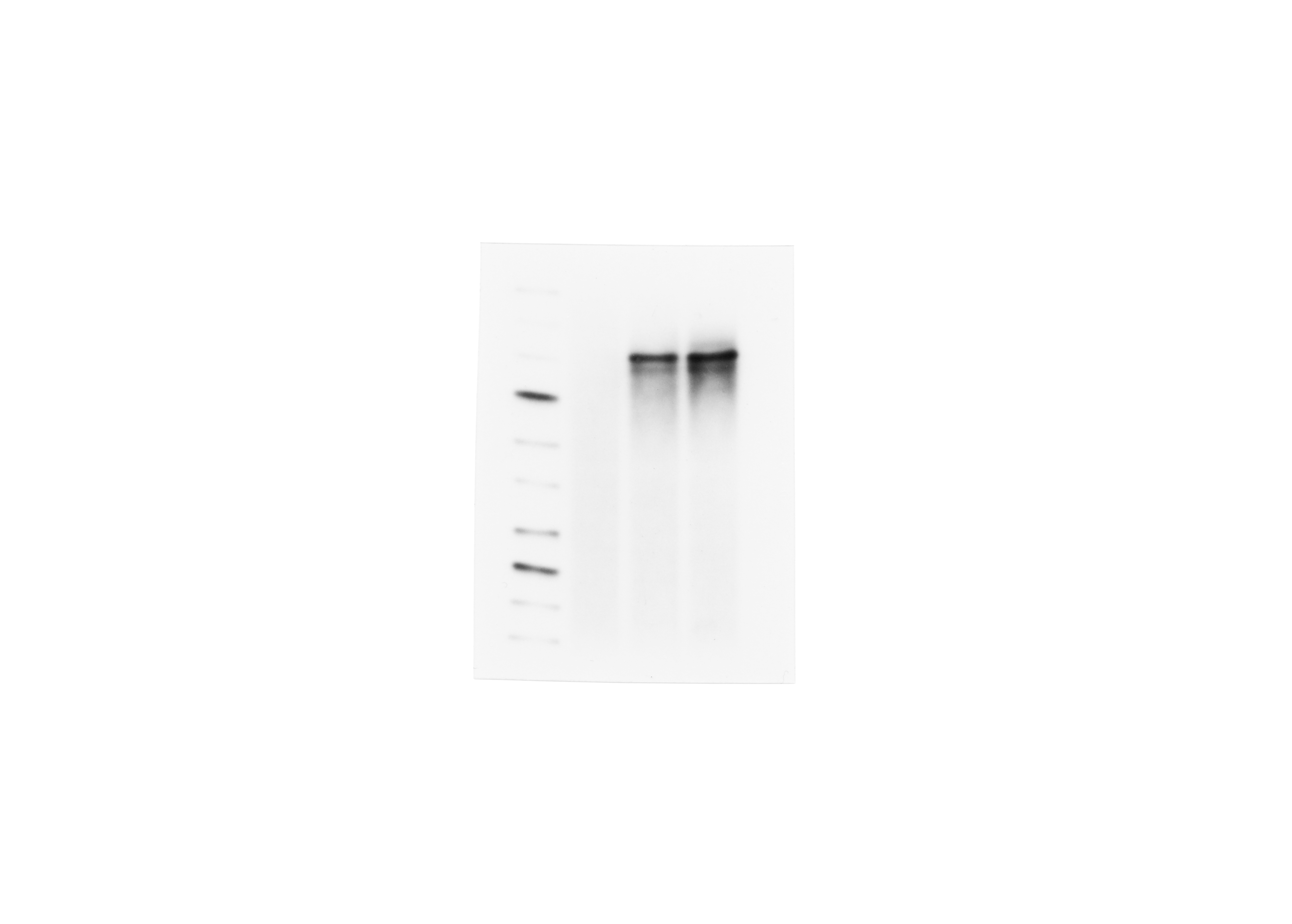

Supplement: Supplementary file 1 — Supplementary Material 1. [file 12885_2026_15659_MOESM1_ESM.zip › original westernblot data/3/Fig10B-ltch.tif]

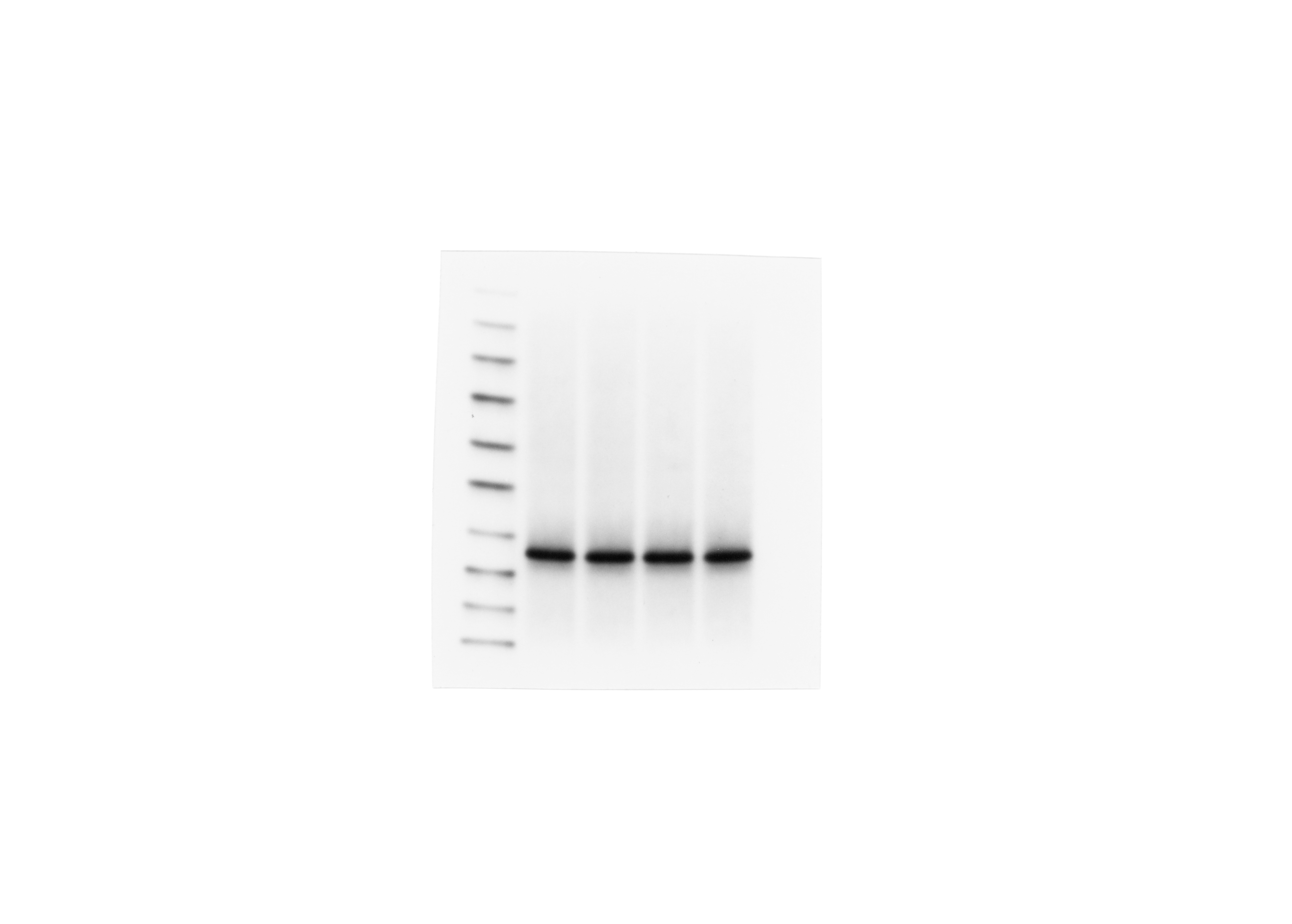

Supplement: Supplementary file 1 — Supplementary Material 1. [file 12885_2026_15659_MOESM1_ESM.zip › original westernblot data/4/Fig10c1-GAPDH.tif]

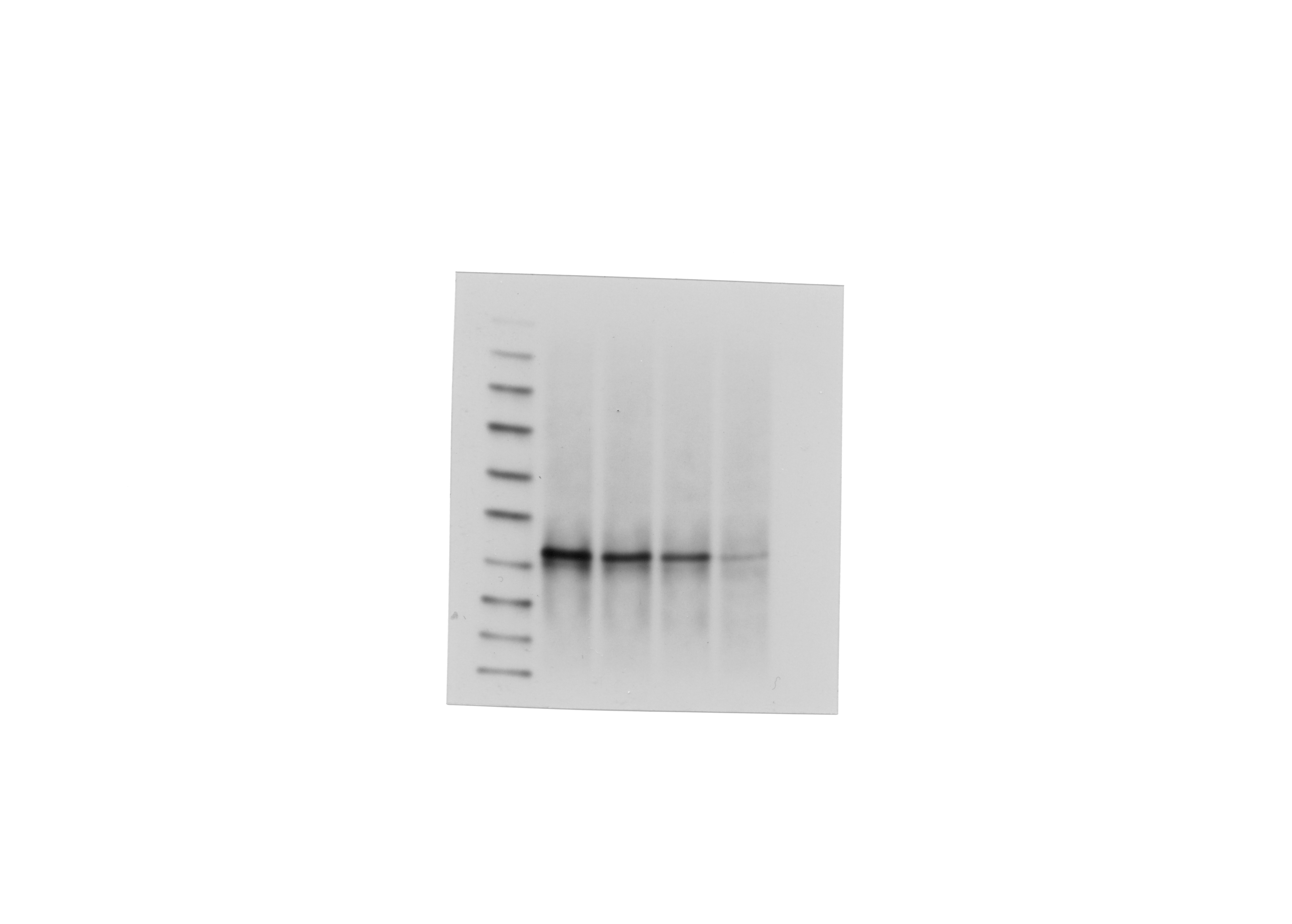

Supplement: Supplementary file 1 — Supplementary Material 1. [file 12885_2026_15659_MOESM1_ESM.zip › original westernblot data/4/Fig10c1-Jun.tif]

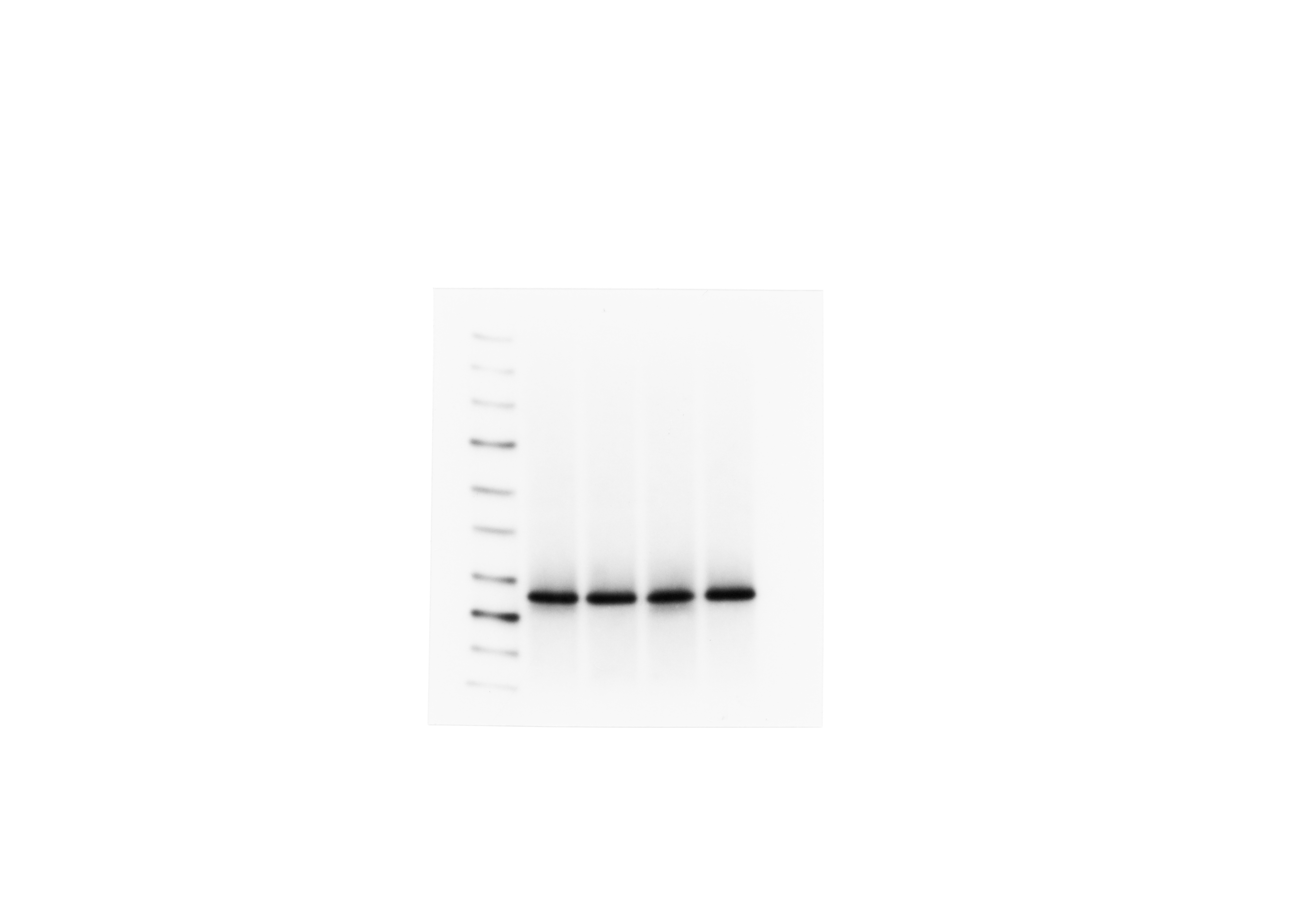

Supplement: Supplementary file 1 — Supplementary Material 1. [file 12885_2026_15659_MOESM1_ESM.zip › original westernblot data/5/Fig10c2-GAPDH.tif]

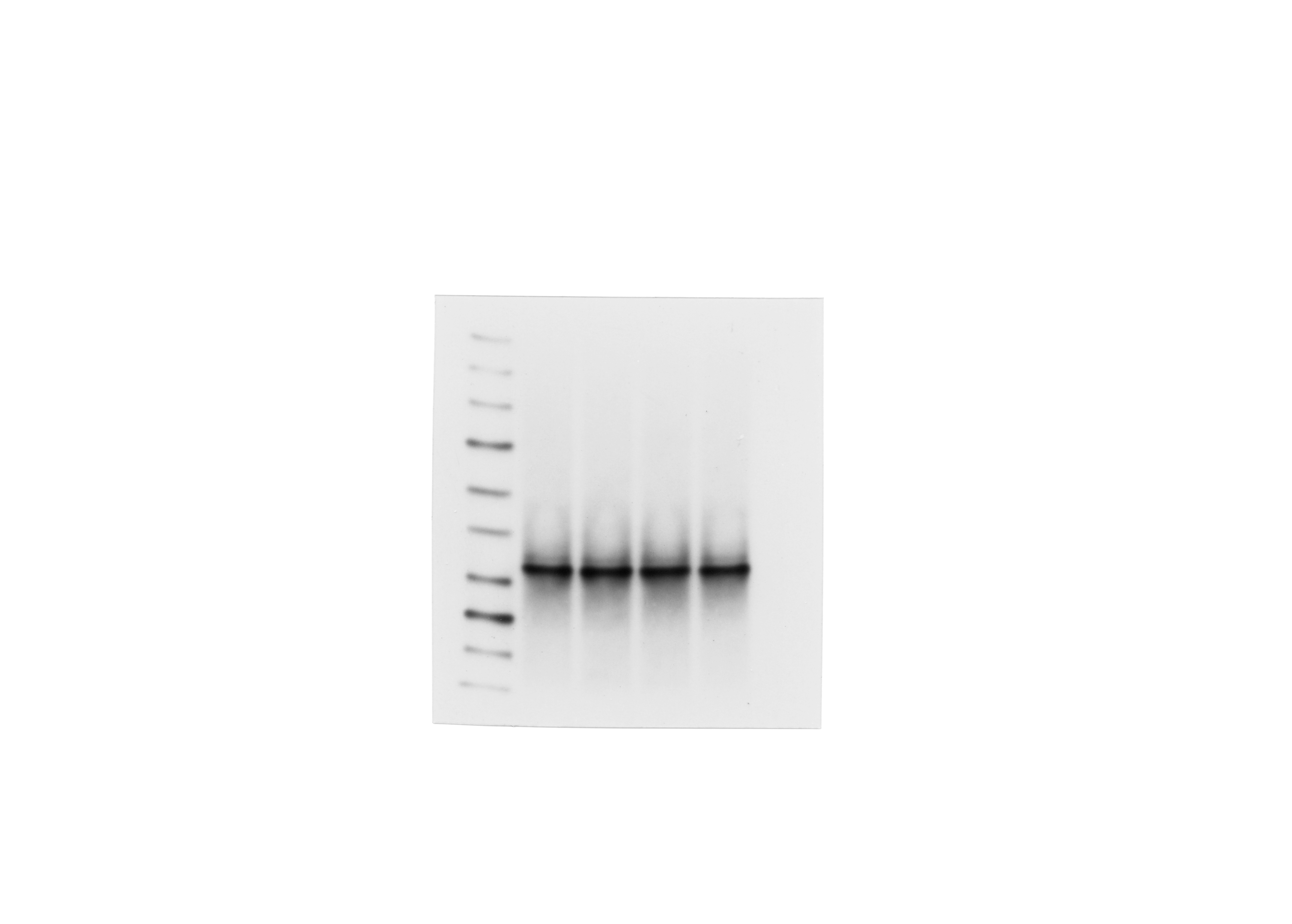

Supplement: Supplementary file 1 — Supplementary Material 1. [file 12885_2026_15659_MOESM1_ESM.zip › original westernblot data/5/Fig10c2-Jun.tif]

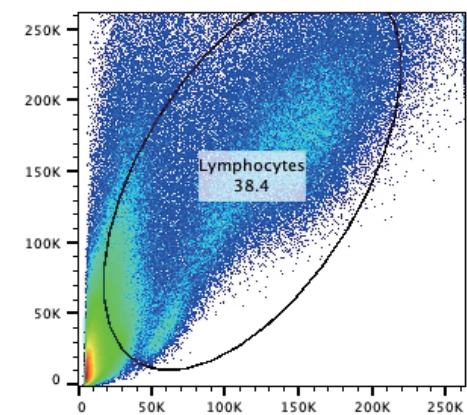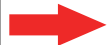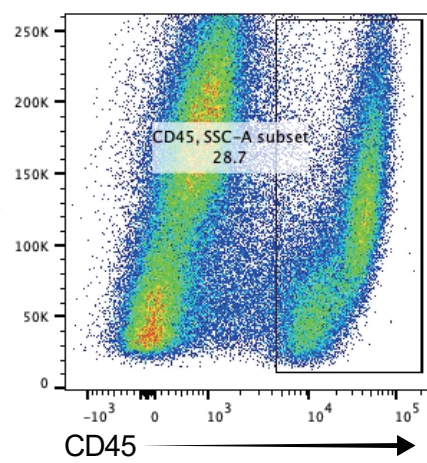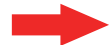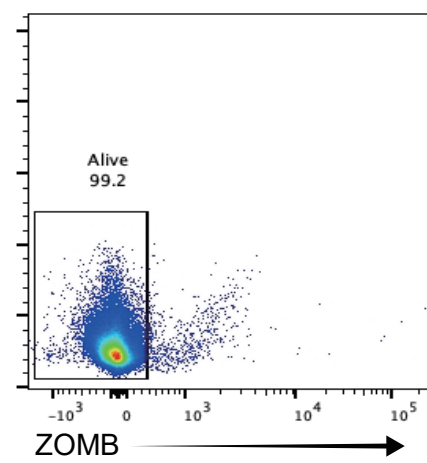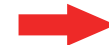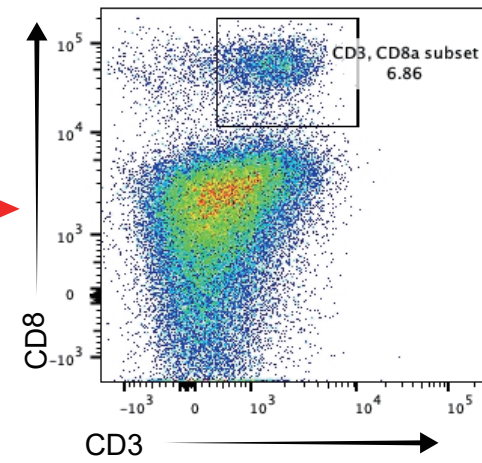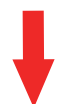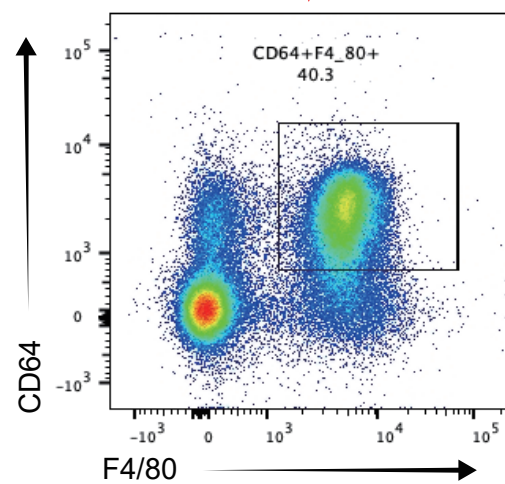

Supplement: Supplementary file 2 — Supplementary Material 2. [file 12885_2026_15659_MOESM2_ESM.pdf]
